# Beyond bacilli: integrating the microbiome into the TB research agenda

**DOI:** 10.1080/19490976.2026.2638004

**Published:** 2026-03-04

**Authors:** Edson Mambuque, Ana del Amo-de Palacios, Samuel G. Huete, Charissa C. Marsh, Grant Theron, Alberto L. García-Basteiro, Sergio Serrano-Villar

**Affiliations:** a Centro de Investigação em Saúde de Manhiça (CISM), Maputo, Mozambique; b Barcelona Institute for Global Health (ISGlobal), Hospital Clinic, Universitat de Barcelona, Barcelona, Spain; c Department of Infectious Diseases, Hospital Ramón y Cajal, IRYCIS, Madrid, Spain; d Carlos III Research Institute, Madrid, Spain; e DSI-NRF Centre of Excellence for Biomedical Tuberculosis Research, SAMRC Centre for Molecular and Cellular Biology, Division of Molecular Biology and Human Genetics, Faculty of Medicine and Health Sciences, Stellenbosch University, Cape Town, South Africa; f Department of Biomedical Sciences, African Microbiome Institute, Division of Molecular Biology and Human Genetics, Faculty of Medicine and Health Sciences, Stellenbosch University, Cape Town, South Africa; g Life Science Campus, Universidad Antonio de Nebrija, Madrid, Spain

**Keywords:** Microbiome, tuberculosis, methodological standardization, confounder control, mechanistic studies

## Abstract

Tuberculosis (TB) remains a leading infectious killer, with growing evidence that the human microbiome—particularly in the gut and lungs—shapes susceptibility, progression, and treatment outcomes. Over the past decade, studies have reported that TB-associated dysbiosis, which is more common in the gut than in the lung, is often marked by the loss of short-chain fatty acid–producing taxa and the expansion of opportunistic microbes. However, findings are frequently confounded by diet, antibiotic exposure, comorbidities, geography, and methodological variability. Most research has relied on compositional profiling, offering limited insight into functional mechanisms. This narrative review synthesizes recent evidence, emphasizing the need to integrate multiomics approaches—metagenomics, metatranscriptomics, and metabolomics—and experimental validation to uncover causal links between microbiome alterations and TB pathogenesis or therapy response. We discuss potential clinical applications, including microbiome-based diagnostics (such as stool-based microbial or metabolite signatures for TB risk stratification), prognostic indicators (such as gut microbiome recovery predicting immune normalization during therapy), and adjunctive interventions (including microbiome-derived products to reduce drug-induced liver injury or fecal microbiota transplantation, which has been shown to be safe in people with HIV on stable ART) to mitigate drug toxicity or enhance immune recovery. Key priorities include methodological standardization, confounder control, mechanistic studies, and the inclusion of high-burden settings. By moving beyond descriptive surveys toward functional, translational research, integrating insights from different microbiome methods into TB prevention, diagnosis, and treatment could redefine the clinical research agenda and open new avenues for precision medicine in this global disease.

## Introduction

Tuberculosis (TB), caused by *Mycobacterium tuberculosis* (Mtb), remains a major global killer.^1^ Beyond the lungs, the respiratory and gut microbiomes may influence TB risk, progression, and treatment outcomes.^2^ Yet, major gaps remain in understanding the risk factors driving progression and treatment outcomes, highlighting the importance of investigating additional host and external modulators—such as concomitant infections (e.g., CMV) and the microbiome—to inform personalized TB control strategies. Although factors such as undernutrition, HIV, and diabetes have stronger effects on TB outcomes, emerging evidence suggests that microbiome shifts—though likely smaller in magnitude—can still influence immunity and drug response. Studies suggest that TB and its therapy perturb these communities, while microbiome composition and function can modulate host immunity.^3^ Interest in a “gut–lung axis” is growing, though interpretation is complicated by confounders such as diet, comorbidities, and sampling methods.^2^^,^^4^ Most research has used 16S rRNA surveys, which reveal composition but not function; distinct communities may share capacities, and functional shifts may drive clinical effects.^5^^,^^6^

The TB field is shifting from cataloguing microbial taxa to decoding the functional and clinical relevance of host–microbiome interactions. This narrative review synthesizes emerging evidence, addresses critical methodological pitfalls, and proposes a translational roadmap for integrating microbiome insights into TB care. Unlike previous reviews focused largely on taxonomic surveys, this piece critically integrates recent evidence on key confounders, functional multiomics analyses, and clinical applications, outlining a translational framework to embed the microbiome into the TB diagnostic and therapeutic agenda. We highlight major confounding variables that must be addressed for reproducible results, discuss differences between pulmonary and intestinal microbiomes in TB, and argue for multiomics approaches that move beyond simple compositional analysis.

Importantly, we advocate that major TB drug and vaccine trials routinely incorporate a microbiome readout—or at least biobanking of appropriate samples—as these large-scale, resource-intensive studies offer a unique opportunity to generate high-quality, clinically relevant microbiome data without duplicating effort. We also explore potential clinical applications of microbiome data in TB—from biomarkers of disease or treatment response to adjuvant therapies—and outline key challenges, knowledge gaps, and future research directions needed to translate microbiome insights into improved TB care.

## Overview of microbiome research in TB

TB–microbiome research has grown rapidly, examining both lung and gut communities. Pulmonary findings, often from sputum as a proxy, are inconsistent: some report no diversity differences,^7^ others note increased *Streptococcus* or *Haemophilus* in TB.^8^ A key contributor to this inconsistency is the use of sputum, which is a heterogeneous mixture of upper and lower airway microbiota. A multicenter study found no universal lung signature, with variation driven by geography and individuals.^9^ Gut studies are more consistent: TB has been associated with depletion of beneficial short-chain fatty acid (SCFA) producers (such as certain Clostridiales), and enrichment of opportunistic pro-inflammatory taxa such as *Enterobacteriaceae*, *Streptococcus*, and *Enterococcus.*^4^ In one cohort, healthy household contacts had more *Prevotella* and *Bifidobacterium*, whereas people with TB had more butyrate-producers like *Faecalibacterium* and *Roseburia.*^5^ Shotgun metagenomic analyses revealed a reduced genetic capacity for vitamin and amino acid biosynthesis and an increased abundance of genes involved in short-chain fatty acid (SCFA) pathways, supporting the hypothesis—yet to be functionally validated—that TB-associated dysbiosis may promote an anti-inflammatory milieu affecting host immunity.^5^ However, even in gut studies, the magnitude and direction of specific taxa-level associations can vary with undernutrition, HIV/diabetes comorbidity, antecedent antibiotics, and local diet—factors that are unevenly measured and adjusted across cohorts.

Anti-TB therapy itself is a major disruptor of the microbiome. Standard regimens rapidly alter gut communities, with marked loss of SCFA-producing *Firmicutes* and expansion of *Bacteroides* within 1–2 weeks.^10^ One longitudinal study showed that while active TB itself led to only a minor decrease in gut α-diversity, initiating treatment caused a profound dysbiosis with >50% of the gut bacterial taxa changing in relative abundance,^11^ while others find minimal changes,^9^ highlighting contextual and methodological influences. However, the majority of human studies focus on short-term changes during the intensive phase of therapy, with follow-up often limited to weeks or a few months, limiting inference about microbiome recovery or persistence beyond early treatment.

Overall, TB and its treatment seem to disrupt both the gut microbiota and airway microbiota, but patterns vary by study and population. Across studies, many reported that pulmonary associations attenuate after accounting for key confounders (including geography, HIV status, sampling approach, and contamination control), contributing to the limited reproducibility of “universal” lung signatures. Consequently, pulmonary microbiome signals in TB should be interpreted primarily as context-dependent until they are validated in well-controlled, multisite cohorts. Recent work has tested whether these shifts influence outcomes, with evidence from human, animal, and Mendelian randomization studies suggesting that the gut microbiota composition can affect TB risk—protective taxa such as *Bacillales* and risk-associated *Bacteroidaceae* have been identified.^12^ These insights are driving interest in microbiome-targeted TB prevention and therapy, despite remaining challenges and confounders. An overview of the current evidence, highlighting areas of convergence and heterogeneity across pulmonary and intestinal studies, together with the major design and methodological drivers of divergence, is summarized in Tables 1 and 2.

**Table 1.** Key confounding factors in TB–microbiome studies.

Confounding factor	Impact on microbiome and considerations in TB studies	References
Diet and Nutrition	Diet shapes gut microbiota composition People with TB often malnourished or with distinct diets. Differences in fiber intake and food diversity can drive microbiome changes unrelated to TB. Controls must be matched for diet or nutritional status to avoid confounding. Malnutrition itself alters immunity and microbiota, potentially influencing TB outcomes.	Morgan et al.^4^
Antibiotic use (incl. TB drugs)	Past or concurrent antibiotics can drastically alter microbiota. TB therapy (prolonged multidrug regimen) causes loss of commensals and overgrowth of others. Microbiome samples taken after treatment may reflect drug effects rather than TB. It is necessary to sample before treatment or include untreated TB and control groups.	Hu et al.^10^ Sala et al.^9^ Wood et al.^2^
Comorbidities (HIV, diabetes, etc.)	Conditions like HIV and diabetes independently affect the microbiome and immune response. HIV coinfection in TB is associated with distinct sputum microbiota (e.g., higher viral load in lung secretions). Diabetic people with TB show different gut microbiome shifts than non-diabetic People with TB. These factors must be stratified or adjusted for; otherwise, microbiome differences may actually be due to the comorbidity.	Morgan et al.^4^ Ticlla et al.^13^
Geography & environment	Regional and environmental differences (urban vs. rural, sanitation, climate) strongly influence baseline microbiome. Multicountry studies found no universal TB microbiome signature, only location-specific patterns. TB prevalence often correlates with low- and middle-income settings, which have different microbiomes from high-income controls. Using local community or household controls is important. Socioeconomic factors (housing, pollution) also act as confounders.	Morgan et al.^4^ Sala et al.^9^
Sampling method/site	Differences in sample type between People with TB and controls can confound results. Control group selection (community vs. household vs. sick controls) strongly influences observed microbiome differences. For lung studies, TB cases produce sputum while controls may give throat swabs—oral flora in swabs can differ from sputum. Sputum passes through the mouth, picking up oral bacteria; thus, some “lung” differences might just reflect oral contamination. Consistent sampling (e.g., using comparable upper airway samples in both groups) or careful interpretation is needed. Similarly, stool vs. mucosal biopsies in gut studies can yield different community profiles.	Botero et al.^14^
Low biomass & contamination	Pulmonary samples in TB are low biomass and therefore highly susceptible to reagent and environmental contamination, making stringent negative controls and decontamination pipelines essential for valid inference. DNA from laboratory reagents or the environment can skew results if not controlled, especially in studies that do not include negative controls. Meticulous lab practice (blank controls, decontamination) and analytical filtering are required to ensure true TB-associated microbes are identified.	Naidoo et al.^15^
Sequencing/analysis differences	Variations in DNA extraction kits, 16S rRNA primers, sequencing platform, and bioinformatics can introduce biases. If cases and controls are processed in different batches or with different protocols, observed microbiome differences may be artifactual (e.g., a given primer might under-detect *Mycobacterium tuberculosis* in cases, or a bioinformatic pipeline might misclassify taxa in one group). Standardizing methods and including batch as a covariate in analysis help mitigate this confounder.	Mori et al.^16^

## Major confounding factors in TB–microbiome studies

**Figure 1.**
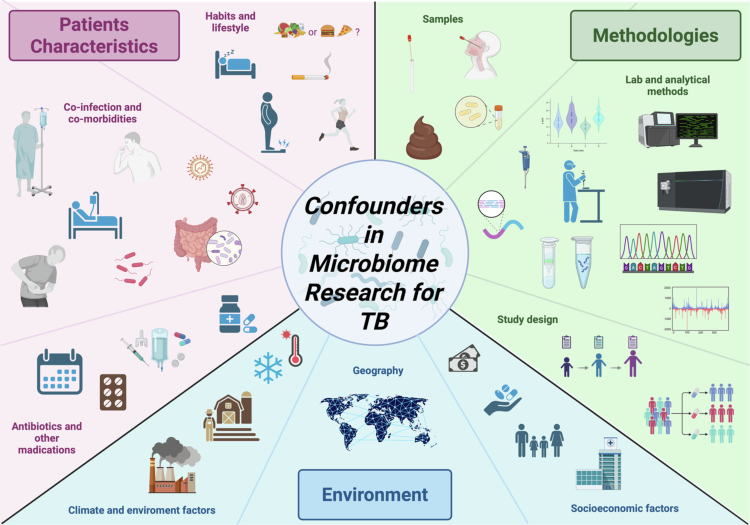
Overview of Confounders in Microbiome Research for TB. These include: (1) Patient characteristics, such as coinfections (e.g., HIV, helminths), comorbidities (e.g., diabetes), antibiotic use, nutrition, and lifestyle; (2) Environmental and contextual factors, including geography, sanitation, climate, and socioeconomic status; and (3) Methodological variables, encompassing sample type, laboratory protocols, sequencing platforms, and study design. Some confounders (e.g., HIV, diabetes, malnutrition, antibiotic use, and socioeconomic status) are particularly important because they can influence both the microbiome and TB susceptibility or progression directly. These dual-impact factors must be carefully accounted for to avoid spurious associations and ensure accurate interpretation and reproducibility of results.

**Figure 2.**
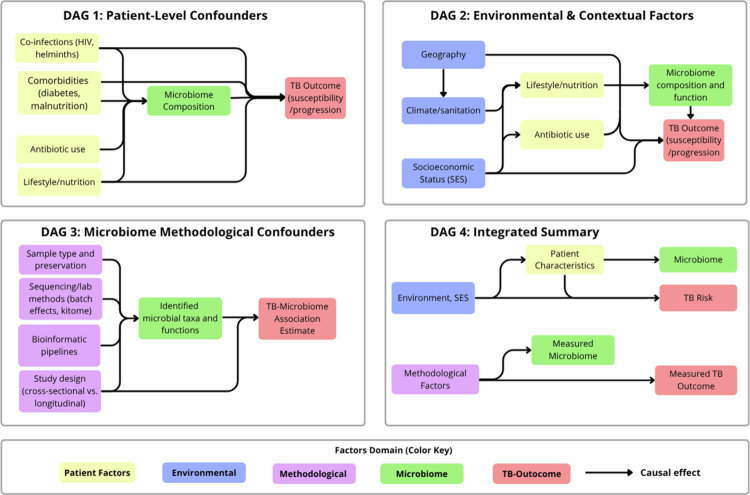
Directed Acyclic Graphs (DAGs) illustrating sources of confounding in microbiome–tuberculosis (TB) association studies. DAG 1–3 depict distinct confounding domains: patient-level (DAG 1), environmental/contextual (DAG 2), and methodological (DAG 3). Each includes variables that may influence both the microbiome and TB outcomes, potentially biasing the observed associations. DAG 4 integrates these domains into a unified causal framework. Node colors denote variable categories. Arrows indicate hypothesized causal relationships.

Low microbial biomass in lung samples represents one of the most critical and underappreciated sources of bias in TB microbiome research. The low agreement among studies exploring the role of the microbiota in human disease remains a major barrier to establishing causal relationships between host-associated microbes and pathology. These discrepancies often arise from uncontrolled confounding factors that influence microbiota composition independently of disease status.^17^ Key confounding factors in TB–microbiome studies include patient-specific variables (e.g., diet, coinfections and comorbidities, medications), environmental and sociocultural factors, and technical and methodological variables. Table 1 and Figure 1 summarize major confounders and their relevance. Figure 2 illustrates these factors using directed acyclic graphs (DAGs) to highlight how different domains—patient, environmental, and methodological—may introduce bias in observed microbiome–TB associations. Not all of these have been conclusively demonstrated as confounders in TB cohorts, but they are plausible sources of variability given evidence from other conditions and some emerging TB-specific studies. Moreover, certain “confounders”, such as poor diet or HIV coinfection, are themselves strong causal risk factors for TB; in these cases, microbiome changes may reflect relevant host‒pathogen interactions rather than noise.

For diagnostic applications, associations between microbiome features and TB may still be clinically useful even if causality is complex. In contrast, mechanistic inference requires explicit control of confounding factors. Notably, only a limited subset of reported TB–microbiome associations have been shown to persist after accounting for major covariates (e.g., pathogen burden/clearance, HIV status, and geography), whereas many pulmonary signatures remain inconsistent across settings (Table 2). While complete control is rarely feasible in human cohorts given the diversity of TB presentations and populations, strategies such as careful case–control matching, stratified analyses, multivariable adjustment, and replication across independent cohorts can help mitigate bias and strengthen the robustness of findings.^16^

**Table 2.** Summary of main findings: pulmonary vs. gut microbiome in TB.

Domain	Pulmonary microbiome (TB vs. healthy)	Intestinal microbiome (TB vs. healthy)
Diversity	Often reduced; low-diversity communities dominated by oral commensals.	Consistently reduced α-diversity; loss of beneficial fermenters.
Taxonomic shifts	No universal TB signature—high inter-person variability and strong confounding by geography, HIV status, sampling site/type, and contamination control. ↑ *Streptococcus*, *Pseudomonas*, *Haemophilus*; ↓ obligate anaerobes. Oral contamination is common. Some TB cases resemble controls.	Convergence on ↓ SCFA producers (*Faecalibacterium*, *Roseburia*) and ↑ *Escherichia*/*Shigella*, *Enterococcus* *Prevotella* often depleted. Taxa-level effects vary by undernutrition, comorbidities, antibiotics, and geography.
Functional changes	Limited data. Oral flora may obscure true lung microbiota signals.	↓ Vitamin biosynthesis; ↑ Metabolism of host-derived compounds (e.g., bile acids, propionate). SCFA balance may shift and affect immune regulation.
Treatment effects	Mixed: some studies report ↓ diversity post-treatment; others show minimal impact. Changes were subtle and confounded by the oral microbiome.	Rapid and marked dysbiosis within days of therapy. ↓ *Firmicutes*, ↑ *Bacteroides*/*Proteobacteria*. Incomplete recovery posttreatment. Long-term (>6–12 months) outcomes largely uncharacterized.
Comorbidities	TB/HIV patients had sputum dominated by commensal viruses (Torque teno, EBV) not seen in HIV-/TB, indicating coinfection alters the lung microbiome.	Risk factors for TB (HIV, malnutrition, diabetes, geography, age) affect the gut microbiome.
Representative studies	Cheung et al.^7^ Ticlla et al.^13^ Sala et al.^9^	Ding et al.^18^ Maji et al.^5^ Morgan et al.^4^ Hu et al.^10^ Pei et al.^19^

### Patient diet and nutritional status

Nutritional status profoundly shapes the gut microbiome and immune function.^20-22^ TB often afflicts populations with high rates of undernutrition and food insecurity. Malnutrition itself is a known risk factor for active TB progression and is associated with microbiome alterations (such as loss of fiber-degrading commensals) that could confound comparisons.^23^ Dietary patterns differ across regions and between cases/controls; for example, a study in West Africa noted that healthy controls from different households displayed substantial microbiome variation owing to differences in diet and socioeconomic status, limiting their utility as a single reference group.^4^ However, even when household contacts are used, studies rarely account for shared exposures or subclinical immune alterations related to recent *M. tuberculosis* contact, underscoring the need to interpret comparator-dependent microbiome differences with caution. Ideally, controls should be well matched for diet and nutritional status, or analyses should adjust for body mass index and diet-related metadata.^24^ Some studies use household contacts of people with TB as controls to minimize differences in diet and environment,^4^ while others have argued that “sick controls” with non-TB lung disease may, in some contexts, provide a more appropriate comparison group.

### Antibiotic exposures (including TB medications)

Antibiotic use is a potent modulator of microbiome composition. Many people with TB have a history of broad-spectrum antibiotic use (for other infections or misdiagnosis) or are started on empiric antibiotics when TB is suspected, which can significantly alter the microbiota before sampling.^2^ More obviously, all people with TB receive prolonged multidrug therapy—most commonly the standard first-line regimen comprising isoniazid, rifampicin, pyrazinamide, and ethambutol (HRZE)—which itself causes microbiome disruption.^10^ If microbiome samples are taken during or after treatment, it becomes difficult to disentangle the effects of the infection from the effects of the drugs. For example, one study found that several gut bacterial genera decreased sharply within a week of TB treatment initiation, which is consistent with the rapid loss of susceptible commensals after HRZE exposure.^10^ If microbiome samples are collected during or after treatment, it becomes difficult to disentangle the effects of the infection from the effects of the drugs. Without proper control, one might incorrectly attribute this loss to TB disease when it is drug-induced.

Importantly, the nature and extent of microbiome perturbation vary not only with the timing of sampling but also with regimen composition and treatment duration, as different drug combinations exert distinct ecological pressures. The relative contribution of individual drugs remains incompletely defined; however, rifampicin's broad antibacterial activity beyond *M. tuberculosis* makes it a biologically plausible contributor to treatment-associated dysbiosis. At a mechanistic level, these alterations are thought to arise through direct suppression of susceptible commensal taxa, followed by ecological niche expansion of intrinsically resistant or tolerant organisms, with additional indirect host-mediated effects. In the respiratory tract, where the baseline microbial biomass is low, even antibiotics not primarily targeting Mtb (e.g., fluoroquinolones or macrolides) can markedly reduce diversity. A 2019 Lancet review emphasized that TB is typically treated with prolonged antibiotics and microbiome composition is often measured at sites of low microbial biomass, meaning that many detected microbiome shifts could be drug effects or contamination.^15^ Importantly, microbiome disruption is not limited to broad-spectrum drugs: even agents considered narrow-spectrum for *M. tuberculosis*, such as isoniazid, have been associated with gut microbial changes, suggesting off-target or indirect mechanisms that remain poorly understood.^25^

### Coinfections and comorbidities

TB frequently co-occurs with other diseases that impact the microbiome. Notably, HIV coinfection is common in TB and causes immunodeficiency, which can alter the microbiota composition (and increase infection by normally latent microbes). In one study, sputum from TB/HIV coinfected patients had a higher prevalence of certain viruses (e.g., EBV, anellovirus) dominating the microbiome,^13^ presumably reflecting HIV-related immune changes rather than TB. HIV, by itself, has been linked to dysbiosis of the gut^26^^,^^27^ and lung microbiome,^28^ so comparing HIV-positive TB cases to HIV-negative controls would be confounded.

Helminth infections (and other gut parasitoses) are also highly prevalent in TB-endemic settings and can reshape the gut microbiome while skewing host immunity toward Th2/Treg phenotypes;^29^ these effects may modulate TB susceptibility and responses and have even been argued to influence BCG effectiveness.^30^

Diabetes mellitus is another major TB risk factor; diabetic patients often have a distinct gut microbiome (e.g., lower butyrate producers, higher opportunists) even without TB. A 2023 study explicitly examined people with TB with and without diabetes and found that TB was associated with gut dysbiosis, but the TB-DM subgroup had additional alterations, notably increased *Bacteroides* and *Blautia.*^4^ However, it remains unclear whether these differences are driven by diabetes itself, TB, or their combined effects, underscoring the challenge of attributing microbiome shifts to a single condition in comorbid populations.

Other comorbidities to consider include chronic liver disease, smoking-related lung disease, and helminth or other chronic infections endemic in some TB populations. Coinfections and host variables such as parasitic infections have been shown to substantially influence the gut microbiome composition and host immune tone and to act as major confounders in human microbiome–disease association studies, particularly in low- and middle-income settings.^17^ Helminth infections, in particular, can substantially reshape the gut microbiome and skew host immunity toward regulatory or Th2-biased responses, with potential implications for TB susceptibility and immune responses.^29^^,^^30^ Accordingly, future studies should collect helminth exposure/treatment data (e.g., stool antigen/PCR, deworming status) or at least store sufficient specimens for retrospective testing and consider stratification by helminth infection, alongside careful matching of HIV and diabetes, or inclusion of these variables in multivariable models to reduce bias and clarify microbiome–TB relationships.^16^^,^^17^

### Geography and environment

The microbiome varies significantly by geography, climate, and lifestyle (urban vs. rural, etc.). People with TB in different regions may have different baseline microbiomes simply because of their environment and culture, not the disease. A multicenter study analysis sputum samples found location-specific signatures, with no universal pattern across sites; a microbial signature seen in Bangladesh was not seen in Switzerland, for example.^9^ Using household or community controls and adjusting for geography can help control for confounding, well-designed multisite studies with standardized protocols and matched controls can also enhance generalizability and help identify microbiome signatures applicable across diverse settings.

Socioeconomic factors (sanitation, crowding, and diet diversity) also confound microbiome findings. One approach is to use household or community controls who share the environment with TB cases.^4^ Another approach is to include geography as a covariate in analysis or focus within a single homogeneous population to reduce environmental variation. Seasonality can even play a role. In a multiomics study in 105 individuals over 4 y in California, the human nasal and gut microbiomes exhibited significant seasonal variation, with greater shifts observed in nasal taxa, and specific gut microbes such as *Holdemania*, *Oscillibacter*, and *Firmicutes.*^31^ That said, in TB-specific cohorts, the effect of seasonality on microbiome composition appeared relatively small compared with that of other drivers, such as geography, host comorbidities, or antibiotic exposure—though TB incidence itself also shows seasonal variation, which may complicate interpretation.^32^ Accordingly, if samples from people with TB were collected in a different season than controls, seasonal diet changes could alter the gut microbiota.

### Sampling site and method

How and from where samples are obtained is a critical methodological factor. This is especially pertinent for the lung microbiome. TB lung disease often requires sputum expectoration for microbiological diagnosis; however, sputum is a mix of lower airway secretions and upper airway flora (from the oropharynx) collected during expectoration. Healthy controls generally do not produce sputum, so studies sometimes use throat swabs or oral wash samples for controls. If not carefully considered, this mismatch can confound the results—differences might stem from sample type rather than TB status. A pilot study in patients with TB and controls comparing nasal swabs, throat swabs, and sputum found that oropharyngeal (throat) communities resembled those in sputum, proposing oropharyngeal communities as a surrogate for control sampling.^14^ Nonetheless, subtle differences exist. Thus, intra-study consistency in sampling is key: some studies collect oropharyngeal swabs from both people with TB (in addition to sputum) and controls to allow direct comparison.^14^

Invasive sampling, such as bronchoalveolar lavage (BAL), yields a more direct lower airway sample but is rarely feasible in healthy controls and can itself introduce organisms or select for certain communities (hospital environment, bronchoscope contamination). For the gut microbiome, stool is the standard sample; differences in stool collection/handling (immediate freezing vs. delay, etc.) can introduce variability, but this is mostly offset by its high biomass, and sampling protocols are easier to standardize.^15^^,^^33^

### Laboratory and analytical methods

Microbiome results can be profoundly affected by DNA extraction protocols, sequencing primers, and bioinformatics pipelines. While standard microbiome considerations (e.g., batch/run effects) should be controlled and corrected, our focus is on TB-specific sources of variation that can masquerade as disease signals. Sputum or BAL are particularly prone to contamination from DNA extraction kits or reagents (the so-called “kitome”).^15^ Sputum generally contains higher microbial biomass than BALF and is therefore somewhat less vulnerable to reagent contamination, but it is instead confounded by oral carryover during expectoration. For example, reagent contamination might introduce *Ralstonia* or *Pelomonas* DNA that could be misinterpreted as part of the lung microbiome if appropriate controls (blank extractions) are not included. Furthermore, “background“ DNA from the medical apparatus used during bronchoscopy needs to be accounted for (i.e., including bronchoscope wash controls during DNA extraction). Distinguishing true lung residents from contaminants is a known challenge in pulmonary microbiome studies.^15^ Furthermore, different 16S rRNA gene primers have varying coverage of key taxa and may underdetect important anaerobes, leading to bias.^34^

Without these controls, apparent enrichment of specific taxa in TB lung samples may reflect contamination rather than true biological differences, contributing to the lack of reproducible pulmonary microbiome signatures across studies.

### Study design

Careful cohort design is essential for disentangling disease-specific microbiome changes from confounding influences. Sampling patients before treatment and tracking longitudinal changes can offer more meaningful comparisons. Because nearly half of individuals with Mtb infection remain asymptomatic, it is critical to distinguish between latent infection, subclinical TB, and clinically manifested disease when selecting study populations. The type and stage of disease (e.g., pulmonary vs. extrapulmonary, drug-susceptible vs. drug-resistant) may also strongly influence microbiome composition. In particular, rifampicin-resistant (RR-TB) and multidrug-resistant TB (MDR-TB), which require prolonged treatment with second-line regimens including fluoroquinolones and agents such as bedaquiline, are likely to exert greater and more persistent microbiome disruption than drug-susceptible TB, although direct comparative data across resistance categories remain limited.

An often-underappreciated source of heterogeneity across TB–microbiome studies is the choice of comparator group. Studies have variably used healthy community controls, household contacts of people with TB, or “sick controls” with non-TB lung disease, each introducing distinct interpretive trade-offs. Healthy community controls are frequently mismatched for diet, socioeconomic status, and environmental exposures, potentially exaggerating apparent dysbiosis. Household contacts provide improved environmental and nutritional matching but may share *M. tuberculosis* exposure or early immune perturbations that complicate the attribution to active disease. Conversely, sick controls may help isolate TB-specific effects but introduce alternative inflammatory states or treatment-related confounding factors. Failure to explicitly align control selection with the underlying research question likely contributes to inconsistent findings and represents a key gap in the current literature. The choice of study population should be guided by the following research question: for example, healthy community controls or household contacts are appropriate when studying microbiome correlates of Mtb infection risk; individuals with latent infection provide a comparator for progression studies; and in active disease, the type of TB (e.g., pulmonary vs. extrapulmonary, drug-susceptible vs. MDR) may itself strongly shape the gut or lung microbiome. Standardizing the definition of patient populations across studies is therefore essential. A more informative approach is to compare people with TB with individuals matched on global performance, nutritional status, weight, and other relevant health metrics. Examples of appropriate cases and controls include: (1) people with TB vs. household contacts for transmission and risk studies; (2) people with TB with vs. without comorbidities (e.g., HIV, diabetes) for comorbidity effects; and (3) people with TB pre- vs. post-treatment for therapy-related changes. Matching cases and controls on key variables—such as age, sex, geographic location, diet, and HIV status—enhances comparability and should be prioritized whenever possible.^24^

Longitudinal sampling before, during, and after TB treatment is particularly powerful for distinguishing disease effects from treatment-related changes within the same individual.^10^ Statistical methods, including multivariable modeling and stratification, can adjust for known confounders such as antibiotic use or diabetes, and many of these biostatistical models are now integrated into bioinformatic tools tailored for microbiome analyses, a rapidly evolving field. Examples include ANCOM-BC for differential abundance^35^ and integrative multiomics frameworks such as mixOmics/DIABLO for linking microbiome profiles with host and metabolite data.^36^ However, the field is still evolving in its ability to model complex longitudinal, multiomic datasets—particularly those with irregular sampling or small cohort sizes. Residual confounding from unmeasured factors remains a challenge. The findings must therefore be interpreted with caution and ideally validated in independent cohorts. Integrating variables such as nutritional status, coinfections, and host genetics into TB microbiome studies will substantially improve the biological inferences that can be drawn.^16^ Causal inference methods such as Mendelian randomization offer further tools to assess directionality in microbiome–TB associations, and harmonized reporting standards (e.g., STORMS),^37^ along with replication across diverse populations, are essential for building a robust and generalizable evidence base.

## Pulmonary vs. intestinal microbiome in TB

TB impacts two key anatomical microbiomes—the pulmonary microbiome at the site of infection and the intestinal microbiome, which influences systemic immunity. These compartments differ markedly in their microbial communities under normal conditions, and TB appears to perturb them in distinct ways. The impact of TB on the microbiome is further shaped by disease phenotype, including pulmonary vs. extrapulmonary involvement and drug-susceptible vs. drug-resistant forms, although the available evidence is uneven across these categories. Most human microbiome data are derived from pulmonary, drug-susceptible TB treated with standard first-line regimens, whereas extrapulmonary TB and drug-resistant TB remain underrepresented. Limited site-of-disease studies in extrapulmonary TB (e.g., lymph node or pericardial TB) suggest distinct local microbial and immunological environments, but these findings are not directly comparable to those of sputum- or stool-based microbiome analyses.

With respect to drug resistance, expected differences in microbiome perturbation—particularly in the gut—are largely inferred from prolonged exposure to second-line therapies rather than from direct comparative microbiome data, which remain scarce across resistance categories (RR−, HR−, MDR−, XDR-TB). This imbalance highlights an important gap for future studies integrating disease phenotype, treatment regimen, and anatomical compartment.

Table 2 provides a summary of the main findings reported in each compartment.

The pulmonary microbiome in healthy individuals is a low-biomass community with relatively low diversity, often resembling dispersed oral flora, which increases susceptibility to contamination and further constrains confident biological interpretation. In TB, the lung environment undergoes inflammation, tissue damage (cavities), and antibiotic exposure—all of which can be altered and mediated by microbial inhabitants. However, studies of the TB lung microbiome have reported heterogeneous results. This variability points to the need for a formal systematic review and individual participant data (IPD) meta-analysis of published studies to harmonize methodologies, account for confounders, and identify reproducible lung microbiome signals in TB.

Importantly, most lung studies rely on taxonomic profiling, and the functional consequences of these compositional shifts remain largely untested. Some common observations include an overrepresentation of oral commensal bacteria (e.g., *Streptococcus, Neisseria*, and *Prevotella*) in TB sputum samples, likely due to increased transtracheal migration or impaired clearance.^13^ In some studies, people with TB had higher abundance of gram-negative opportunists such as *Pseudomonas* and *Acinetobacter* in sputum, especially those with lung cavitation or more severe disease (possibly reflecting colonization of damaged airways). On the other hand, at least one study found no significant difference in overall diversity or community composition between TB and control sputum microbiota.^7^

The multicenter analysis by Sala et al. concluded that there was no consistent TB-specific lung microbiome signature across different populations.^9^ They observed that each patient's sputum microbiota was highly individualized and influenced by geography and HIV status more than by TB.

Intriguingly, one cross-sectional study noted an inverse relationship between *Streptococcus* and certain anaerobes in sputum: people with TB whose sputum was dominated by *Streptococcus* had lower overall diversity, whereas those with more anaerobic genera (like *Selenomonas*, *Fusobacterium*) had higher diversity.^13^ This suggests that a balance between different microbial groups (perhaps influenced by oxygen levels or inflammation in lesions) might shape the lung microbiome diversity in TB.

Another observation is that in people with TB coinfected with HIV, the pulmonary microbiome can include a substantial viral component—*Torqueteno* virus and *Epstein–Barr* virus (EBV) were found to sometimes dominate the DNA in sputum of TB/HIV patients.^13^ This raises the question of whether such shifts in the pulmonary virome and broader microbiome could contribute to the increased risk of TB observed in people with HIV, beyond the effects of immune depletion alone.

These findings suggest that several reported pulmonary associations may reflect confounding by host and contextual factors rather than TB disease per se, reinforcing the need for harmonized protocols and carefully matched comparator groups. Differences in comparator groups across studies—particularly the use of unmatched community controls vs. household contacts or alternative respiratory disease controls—likely further contribute to the observed lack of reproducible pulmonary microbiome signatures.

Functionally, most lung microbiome studies have been taxonomic due to low biomass limiting metagenomic sequencing, which is costly. It remains unclear if TB lung dysbiosis (to the extent it exists) has functional consequences for Mtb growth or host immunity in the lung or if it is largely bystander.^38^ Some hypothesize that the loss of beneficial commensals in airways could reduce colonization resistance against pathogens, potentially paving the way for secondary infections or exacerbating inflammation.^39^

In contrast, the intestinal microbiome in TB has shown more consistent patterns of disruption. Numerous studies from different countries (China, India, Africa, etc.) report that gut microbial diversity is reduced in active TB.^18^ One meta-analysis described a TB-associated gut “dysbiosis signature” characterized by depletion of key SCFA-producing families (such as *Ruminococcaceae* and *Lachnospiraceae*) and enrichment of taxa that can be pro-inflammatory or opportunistic (such as *Enterococcus, Streptococcus, Candida* in the mycobiome).^5^ For example, an early Chinese study found people with TB had lower relative abundance of *Bifidobacterium* and *Prevotella* but higher *Clostridium* and *Enterobacteriaceae*, with several inflammation-related functional pathways upregulated in microbiome predictive analysis.^18^ Similarly, a Ghanaian study in 2023 noted that all people with TB (with or without diabetes or HIV) shared a core dysbiosis: increased *Escherichia/Shigella*, *Streptococcus*, *Enterococcus* (potential pathogens) and decreased beneficial butyrate-producers.^4^

The presence of *Akkermansia* (a mucin-degrading genus often linked to gut barrier integrity) was also found to be reduced in some TB cohorts, which might have implications for gut permeability and systemic inflammation.^19^ Gut dysbiosis in TB is not only a consequence of the disease but is exacerbated by TB treatment. Anti-TB therapy causes a rapid drop in commensal *Firmicutes* (like *Faecalibacterium* and *Roseburia*) and often a bloom in *Bacteroides* and other taxa that resist the drug cocktail.^4^ Hu et al. (2019) observed that within one week of starting HRZE therapy, *Firmicutes* abundance plummeted while certain *Bacteroides* OTUs and *Erysipelotrichaceae* spiked, leading to a marked shift in community structure.^10^ Over the course of TB treatment, some recovery of diversity can occur after the intensive phase, but many patients have a persistently altered gut microbiota even at treatment completion.^10^ Mouse models mirror this: conventional mice treated with TB drugs showed lasting depletion of gut microbes, and interestingly, dysbiosis was more severe with certain drug combinations.^19^

Functionally, the gut microbiome of people with TB has been linked to changes in metabolic outputs—for instance, lower production of SCFA like butyrate (due to loss of producers) and possibly altered tryptophan metabolism that could affect immune regulation.^5^ There is emerging evidence that these gut microbiome changes have systemic effects: a study integrating gut microbiome data with blood immune profiles found that the abundance of *Clostridiales* (clusters IV and XIVa) in people with TB correlated with a normalization of peripheral inflammatory markers during treatment.^6^ In other words, as TB treatment progressed, patients whose gut microbiota regained beneficial *Clostridia* saw a stronger resolution of inflammation, independent of pathogen clearance. This points toward a gut‒immune interaction in TB, where the gut microbiome composition can influence systemic immune tone (the concept of the gut‒lung axis) or alternatively reflect a shared underlying cause (such as malnutrition or ART in PWH) affecting the gut and lung microbiotas and host immunity.

In summary, both the pulmonary and intestinal microbiomes in TB show dysbiosis, though gut changes are more consistent across studies. The “gut–lung axis” proposes that the loss of SCFA-producing gut microbes can trigger aberrant lung immune responses, influencing TB progression.^16^ Conversely, TB infection and inflammation may alter the gut ecology via systemic effects, creating a bidirectional relationship. Mendelian randomization suggests that the gut microbiome can causally influence TB risk,^12^ potentially explaining differences in susceptibility beyond established risk factors.

## Composition vs. function: Multiomics approaches

Most early TB–microbiome studies focused on taxonomic composition (e.g., which bacteria increase or decrease in TB) using 16S rRNA gene sequencing. While informative, this approach has inherent limitations. Microbiome function can diverge from composition—different microbial communities may carry out similar functions (functional redundancy), and conversely, similar community compositions can have different metabolic outputs depending on gene expression and the environmental context. In the context of TB, a purely compositional view might tell us, for example, that people with TB have less *Prevotella* and more *Enterococcus* in their gut. However, it does not tell us what that means for the host: Are SCFA levels reduced? Is there accumulation of pro-inflammatory metabolites? Are microbial genes for vitamin K or vitamin B synthesis lost? Such functional insights are critical for understanding how microbiome changes might influence TB disease (or vice versa). Therefore, we are increasingly adopting multiomics approaches—integrating metagenomics, metatranscriptomics, metabolomics, and even host transcriptomics or proteomics—to go beyond simple community profiling.^40^

**Figure 3.**
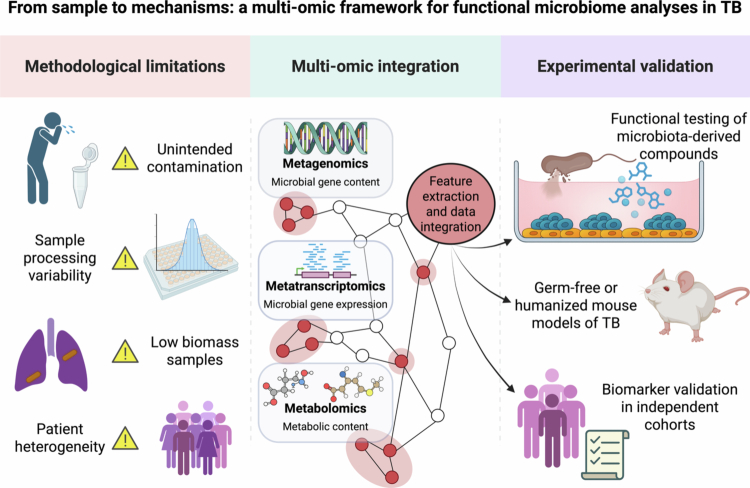
Integrated multiomics and experimental validation pipeline for TB–microbiome research. Microbiome studies in tuberculosis face methodological challenges, including sampling variability, low biomass, and batch effects (left panel). To move beyond descriptive composition, a multiomics approach combines metagenomics, metatranscriptomics, metabolomics, and host omics to uncover functional pathways linking microbiota to TB outcomes (center). These insights must be tested through experimental validation, including mechanistic studies in animal models and host immune assays (right). Together, this framework enables robust causal inference and supports the discovery of translational applications, such as biomarkers or microbiome-based interventions. Arrows do not imply causality unless supported by experimental validation.

A multiomics strategy in TB microbiome research might involve: sequencing the metagenome of patient samples (to catalog microbial genes, including those for metabolic pathways or antibiotic resistance), performing metatranscriptomics (RNA sequencing of the microbiota to determine which genes are actively expressed), and conducting metabolomic analyses of patient blood, stool, or sputum (to measure concentrations of microbial metabolites). These data layers can then be correlated with host data such as cytokine profiles or clinical outcomes. Figure 3 depicts a general pipeline for such an integrative analysis. By combining data, one can identify not just “who is there” but also “what are they doing” and “how it affects the host.”

One example of multiomics insight comes from the integrative study by Wipperman et al. In that work, researchers collected fecal samples, sputum, and blood from people with TB during treatment and applied 16S sequencing for the microbiome, conventional assays for Mtb burden, and RNA-seq for host blood transcriptomes. Using machine learning, they found that changes in the gut microbiome (specifically, increases in Clostridial families and decreases in Proteobacteria) were independently associated with changes in the host peripheral immune transcriptome remained associated with peripheral immune transcriptomic changes after accounting for pathogen clearance, although residual confounding (e.g., diet, comorbidities) cannot be excluded in observational human data.^6^ In other words, as patients got better, certain gut microbes rebounded and correlated with reduced systemic inflammation. This finding suggests a functional link: metabolites from those gut microbes could modulate the immune response to TB treatment. Notably, most longitudinal studies—including those linking microbiome recovery to immune normalization—end follow-up at or before treatment completion, leaving the durability of these associations and their relevance to relapse risk or post-TB lung disease largely unknown.

Complementing this, Naidoo et al. analyzed oral, sputum, and stool microbiota together with host blood transcriptomes in untreated people with presumptive TB. They identified TB-specific microbial relationships, including enrichment of stool anaerobes such as *Erysipelotrichaceae* and *Blautia*, which correlated with host pro-inflammatory pathways (e.g., death receptor and inflammasome signaling), thereby supporting a role of the gut microbiota in shaping TB-associated immune responses even before therapy.^41^ Such conclusions could not be reached from microbiome or host data alone; it was the integration that allowed the authors to propose that “the response to therapy may be a combined effect of pathogen killing and microbiome-driven immunomodulation”.

Another area where multiomics is illuminating is in identifying microbial metabolites or pathways that might influence TB. For instance, metabolomic profiles of people with TB have revealed alterations in tryptophan and bile acid metabolites that could be traced back to microbiome changes.^42^ Some gut bacteria produce indole derivatives that modulate the immune system; a decrease in such bacteria in TB might lead to lower levels of these immunoregulatory molecules, potentially affecting lung granuloma formation. Interestingly, one of these metabolites—indole-3-propionic acid—not only regulates host inflammation but also directly inhibits *M. tuberculosis* by targeting its tryptophan biosynthesis pathway, highlighting a dual host- and pathogen-directed role for microbiome-derived indoles.^43^

Such functional links are typically monitored by integrating metabolomic profiling of host samples with metagenomic and immunological data, enabling correlations between microbial taxa, their metabolic outputs, and host immune responses. Multiomics analysis can link the dots—for example, a reduced abundance of Indolepropionibacter in the gut metagenome could result in lower concentrations of indolepropionic acid in the blood metabolome, which in turn may be associated with heightened inflammation in the host transcriptome. Indeed, an emerging hypothesis is that SCFAs produced by gut microbes (such as butyrate) can suppress inflammation and thus suppress the TB-specific immune response. In mouse models, antibiotics that disrupt the gut microbiota reduce macrophage-inducible C-type lectin (Mincle)-dependent activation of lung dendritic cells, leading to weaker Th1/Th17 responses and higher *M. tuberculosis* burden. Remarkably, these immune defects—and increased susceptibility—can be reversed either by stimulating Mincle with the agonist trehalose-6,6′-dibehenate or by supplementing with *Lactobacillus.*^44^ This kind of mechanistic insight requires combining microbiome data with immunological readouts (a form of cross-omics integration).

​​From a technological standpoint, advances in sequencing and computational biology now make it feasible to perform such integrated analyses. Metagenomic sequencing can detect not only bacteria but also viruses and fungi in TB patient samples—this is important since people with TB often have perturbations in fungal (mycobiome) and viral communities as well.^13^ For example, TB gut dysbiosis includes fungal overgrowth (e.g., *Candida* spp.) in some patients;^5^ metagenomic or internal transcribed spacer (ITS) sequencing can capture these changes. Metatranscriptomics can determine if *M. tuberculosis* itself is active in the microbiome community context—one study identified Mtb RNA transcripts in sputum microbiome data, providing clues about Mtb physiology in patients alongside commensal activity.^45^ Furthermore, shotgun metagenomics allows the assembly of genomes of uncultured organisms that may play roles in TB (For instance, gut bacteria that metabolize TB drugs or modify host metabolites). Multiomics approaches have identified microbial genes associated with drug metabolism; e.g., certain gut microbes harbor β-glucuronidase enzymes that can reactivate toxic TB drug metabolites in the gut,^46^ potentially contributing to drug-induced liver injury.^47^ By sequencing stool metagenomes of people with TB with vs. without liver toxicity, one study found differences in these functional genes^19^—information that 16S alone would have missed.

To manage the complexity of multiomics data, new computational pipelines (such as the gNOMO of the MIXOMICS tool for host–microbiome multiomics integration^48^^,^^49^ and network analysis methods are being employed. These can construct host‒microbe interaction networks, identify cooccurring patterns, and pinpoint candidate “hub” metabolites or genes that link the microbiome and host responses.

In summary, moving from composition to function via multiomics is critical in TB microbiome research. It will help us to:

• Determine which microbial changes have functional importance (for example, loss of a microbe that produces an antimycobacterial compound is more significant than loss of one that does not).
• Identify microbial metabolites or gene pathways that interact with the host during TB.
• Discover potential biomarkers of disease or treatment response that are metabolite-based (which might be more clinically practical than sequencing every patient's microbiome).
• Understand the mechanism of how microbiome alterations might affect TB immunity or drug response.

Recent studies integrating multiomics are already yielding richer hypotheses—such as the idea that restoring certain gut microbial functions could enhance TB treatment response.^6^ However, these approaches also come with challenges: increased cost, analytic complexity, and the need for interdisciplinary expertise (microbiology, immunology, systems biology). Despite these challenges, the consensus in the field is that a multiomics lens is needed to fully grasp the TB–microbiome interplay.^40^ As such, we are beginning to see a paradigm shift from simple case‒control 16S surveys to longitudinal, multilayered studies that can inform not just if the microbiome is different in TB but also *how* and *so what*.

## Potential clinical applications

**Figure 4.**
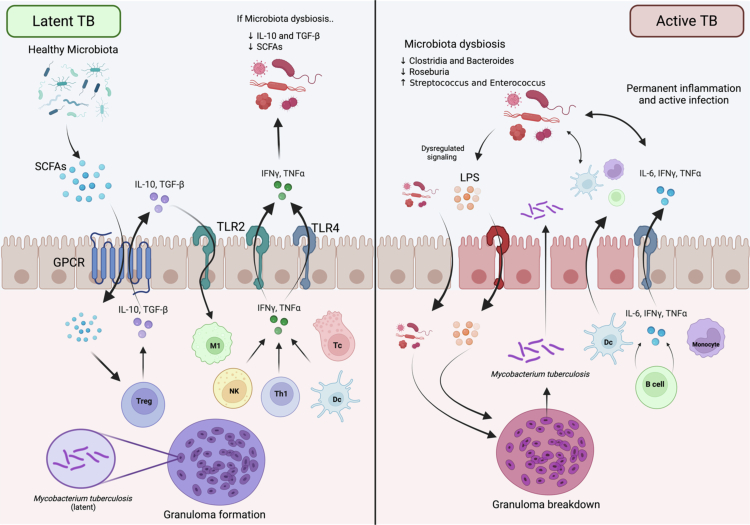
Microbiome-driven modulation of host immunity in tuberculosis: pathways to latency or active disease. This figure illustrates two contrasting immunological outcomes in tuberculosis—immune containment vs. disease progression—driven by gut microbiome composition and function. *Left panel (latent TB):* a balanced and diverse gut microbiota supports homeostatic immunity through the production of short-chain fatty acids (SCFAs), including butyrate and propionate. These metabolites enhance regulatory T cell (Treg) function and IL-10 secretion via G protein-coupled receptor (GPCR) signaling, promote gut epithelial integrity, and prevent microbial translocation. Commensal-driven modulation of Toll-like receptor (TLR) signaling (TLR2/TLR4) ensures appropriate activation of IFN-γ and TNF-α responses, sustaining granuloma formation and stability. These mechanisms collectively support the containment of *Mycobacterium tuberculosis* within granulomas, maintaining a latent infection state. *Right panel (Active TB):* microbiome dysbiosis—resulting from antibiotic exposure, HIV infection, or metabolic comorbidities—alters SCFA-producing species (e.g., *Clostridia*, *Roseburia*, *Bacteroides fragilis*) and increases the abundance of potentially detrimental taxa (e.g., *Enterococcus, Escherichia, Shigella*, and *Enterobacteriaceae*). SCFA depletion weakens epithelial barrier integrity, facilitating the translocation of microbial products such as lipopolysaccharide (LPS) and elevating the levels of LPS-binding protein (LBP). This systemic microbial translocation contributes to dysregulated TLR signaling and cytokine production toward an inflammatory profile (↑ IL-6, ↓ IL-10, and ↓ IFN-γ), impairing macrophage activation and granuloma integrity. The resulting immune dysregulation promotes breakdown of granuloma structures and progression to active TB disease.

Understanding the microbiome's role in TB offers tangible opportunities to improve care. Figure 4 illustrates how the gut microbiota can sustain Mtb infection via SCFA-mediated Treg activation and granuloma stability, whereas dysbiosis—driven by factors such as LPS translocation and cytokine imbalance—can promote progression to active disease. Reliable microbiome patterns or metabolites linked to TB risk, progression, or treatment outcomes could serve as biomarkers or therapeutic targets. If causality is confirmed (e.g., protective commensals), microbiome-based interventions such as probiotics or dietary modulation could complement standard therapy. This section explores three main clinical applications: biomarkers for diagnosis and prognosis, microbiome predictors of treatment response or adverse effects, and targeted therapies to improve outcomes or reduce toxicity.

### Microbiome biomarkers for TB diagnosis and risk stratification

Microbiome composition or metabolite profiles could complement TB diagnosis and risk assessment. Unlike conventional tests that detect pathogens, microbiome biomarkers would use the host's microbial “fingerprint” as an indirect indicator. Distinct gut profiles could enable stool-based screening, though differences between TB infection (TBI) and TB disease remain inconsistent.^9^ A multicenter lung study found no robust pulmonary TB signature,^9^ but machine learning on sputum data achieved moderate discrimination from other lung diseases.^50^

It is important to distinguish between two potential applications: one is the direct detection of Mtb reads or transcripts in metagenomic or metatranscriptomic datasets, which would serve as a pathogen-specific diagnostic; and the other is the identification of TB-associated microbial or metabolite signatures (e.g., shifts in taxa or metabolites) that reflect host–microbiome interactions rather than the presence of Mtb itself. Blood-derived microbial products, such as small RNAs from *M. tuberculosis* or other microbiota,^51^ and serum metabolites—including SCFAs, tryptophan catabolites, and lipids—are under investigation.^52^^,^^53^ Baseline microbiota may also predict TB risk: Mendelian randomization links higher *Bacteroides* and lower *Bacillales* with susceptibility,^12^ ​​supporting potential for microbiome-based risk stratification and targeted preventive or supportive interventions in high-risk or dysbiotic TB subgroups.

### Prognostic indicators and treatment response

Microbiome profiling may help predict TB treatment response or relapse risk. Treatment is lengthy, and patients vary in culture conversion time, relapse rates, and side effects. Early identification of those at risk could guide management. Wipperman et al. reported that gut microbiome recovery paralleled immune normalization,^6^ suggesting that microbiome monitoring as a surrogate for progress. For example, a stool profile after 1 month might reveal a “resilient” vs. “collapsed” microbiota, the latter linked to poor health or persistent inflammation. Persistent dysbiosis after therapy—such as loss of beneficial immunomodulatory microbes—may also increase relapse risk. A cross-sectional Chinese study found recurrent TB cases had higher *Haemophilus* and *Streptococcus* in sputum than new cases,^50^ hinting at a link between microbiome features and chronicity. If validated, microbiome or metabolite biomarkers could inform TB management—For instance, a low “microbiome health index” prompting closer follow-up or targeted adjunctive therapies. An additional hypothesis, put forward by Scriba et al.^54^ is that anti-TB treatment may also deplete commensal NTMs that contribute to maintaining immune responsiveness to Mtb, raising the possibility that therapy itself could inadvertently influence susceptibility to future TB exposures. Extending microbiome follow-up beyond the standard 6-month treatment course represents a major unmet need, particularly to understand long-term immune recovery, relapse susceptibility, and post-TB lung disease.

### Microbiome-targeted adjunct therapies

Perhaps the most promising application is microbiome modulation to improve TB outcomes through probiotics, prebiotics, dietary interventions, or fecal microbiota transplantation (FMT). Evidence from human and animal studies suggests that enhancing beneficial commensals could boost anti-TB immunity, while preventing their loss during treatment may accelerate recovery and reduce side effects. Approaches successful in other conditions, such as probiotics for antibiotic-associated diarrhea or FMT for *Clostridioides difficile* colitis, could be adapted to TB.^55^ Small trials in people with TB have shown that *Lactobacillus* or *Bifidobacterium* supplementation may decrease systemic inflammation and reduce gastrointestinal symptoms.^56^

Addressing TB-associated vitamin B deficiencies via probiotics or fermented foods is another option. Preventing drug-induced liver injury (DILI) is a key target: certain gut bacteria can metabolize isoniazid into hepatotoxins, and patients who developed DILI showed lower *Phascolarctobacterium* and *Akkermansia*, and higher *Bacteroides* and *Enterococcus.*^19^ Patients with DILI had lower baseline *Phascolarctobacterium* and *Akkermansia* (SCFA and mucin-degraders) and higher *Bacteroides* and *Enterococcus*, suggesting that a more proinflammatory gut milieu.^19^ These differences were present within 2–3 weeks of therapy. This raises the possibility that probiotic or dietary measures could prevent DILI—e.g., adding butyrate-producing bacteria or their substrates to maintain intestinal integrity and reduce the translocation of inflammatory microbial products to the liver.^42^ Some groups have proposed administering probiotics alongside TB drugs to decrease the incidence of hepatotoxicity and antibiotic-associated diarrhea.^57^ These insights support a “microbiome stewardship” approach—supporting or restoring gut ecology during and after TB therapy—to increase cure rates, reduce toxicity, and potentially limit post-TB lung damage.

Any microbiome-targeted intervention for TB requires caution. In immunocompromised patients, such as those with HIV, live probiotics may pose an infection risk at very low CD4 counts, and some strains can metabolize TB drugs such as isoniazid or rifampicin, warranting careful selection. Still, potential benefits include faster recovery, fewer side effects, better quality of life, and possibly reduced relapse if protective immunity is supported. Precision medicine could tailor interventions: patients with severe dysbiosis may need intensive probiotic regimens, while those with preserved microbiota might not. Randomized trials are needed to test whether restoring gut microbes via probiotics or FMT reduces inflammation or toxicity.^55^ Early steps include studies combining BCG vaccination with neonatal microbiome profiles to explore probiotic “adjuvants.”

Beyond interventions, microbiome insights could inform public health: integrating nutritional support if diet proves key or screening high-risk populations for predisposing microbiome traits. Notably, the RATIONS cluster-randomized trial in India showed that providing food rations and micronutrients to household contacts reduced incident TB by ~40%–50% over 2 y^58^; embedding the stool microbiome and metabolomic readouts in such large biosocial trials could help explain differential benefits via diet–microbiome–immunity pathways and guide targeted scale-up. Although not yet routine in TB care, the microbiome holds promise for diagnosis, prognosis, and adjunctive therapy, reflecting a shift “beyond bacilli” toward viewing human microbial ecology as both a tool and a target.^57^

## Challenges and future directions

The integration of microbiome research into the TB field, while promising, comes with numerous challenges that must be addressed. In this section, we outline key challenges and propose future research directions to advance our understanding of the TB–microbiome relationship and to eventually translate findings into clinical benefit. Major areas of focus include: resolving inconsistencies across studies through standardization, strengthening causal inference, expanding research to under-studied facets (e.g., mycobiome and pediatric populations) and designing interventional studies. Table 3 summarizes the main barriers currently limiting the translation of TB–microbiome research into clinical benefit and outlines practical, evidence-informed solutions.

**Table 3.** Research priorities and proposed solutions for advancing TB–microbiome research.

Challenge	Description	Proposed Solutions
High inter-study variability	Differences in results due to inconsistent sample collection, sequencing methods, and populations	Adopt standardized protocols for sampling, DNA/RNA extraction, and analysis; follow reporting guidelines (e.g., MIRROR, STORMS); share datasets for harmonized re-analysis
Confounding factors	Patient diet, antibiotics, coinfections (HIV, diabetes), geography, and sampling site all confound microbiome signals	Match cases/controls on key variables (e.g., diet, HIV status), use stratified analysis, collect detailed metadata, and apply multivariate adjustment
Causality unclear	Most human studies are cross-sectional and cannot distinguish cause from consequence	Use longitudinal cohort designs (e.g., household contact follow-up), launch clinical trials (e.g., probiotics), and Mendelian randomization analyses to infer causality
Low biomass & contamination (especially in lung samples)	Risk of detecting reagent or environmental contaminants rather than true microbes in sputum/BAL	Include negative/blank controls, perform decontamination filtering, and interpret low-biomass results cautiously
Limited functional data	16S rRNA sequencing gives taxonomic profiles, but lacks insight into microbial function	Integrate multiomics (metagenomics, metabolomics, and transcriptomics), and link microbial profiles to host immunity and metabolite outputs
Understudied microbial domains	Fungal (mycobiome) and viral (virome) communities are rarely studied, despite relevance in TB and antibiotic use	Include ITS and viral metagenomic sequencing; analyze fungal and viral dynamics alongside bacterial profiles
Neglected populations or TB forms	Pediatric TB, extrapulmonary TB, pregnant women with HIV, and high-risk populations (e.g., malnourished) are underrepresented	Design studies focused on children, intestinal TB, and high-burden settings; include community and nutritional context
Lack of translational studies	Few trials test if microbiome modulation improves TB outcomes or reduces toxicity	Launch randomized trials of microbiome-based adjunct therapies (e.g., probiotics, diet, and FMT); explore post-TB microbiome recovery strategies
Fragmented research landscape	Siloed studies with limited data sharing or cross-disciplinary input	Promote collaborative consortia, build capacity in TB-endemic regions, and support interdisciplinary research (clinical, microbiome, immunology, and systems biology)

### Standardization and methodological rigor

As highlighted, a major challenge is the heterogeneity in methods and populations across studies, which contributes to inconsistent results. Future research should prioritize standardized protocols for sample collection, DNA/RNA extraction, and sequencing when feasible. Efforts such as the NIH's Human Microbiome Project have shown the value of standardizing methods to compare results across sites—a similar approach in TB could enable meta-analyses that identify truly robust microbiome signals. The development of guidelines for TB microbiome studies (e.g., how to handle low-biomass lung samples and recommended confounder data to collect^17^) would help new studies avoid past pitfalls.

Additionally, making datasets (including comprehensive metadata) publicly available for reanalysis can allow us to apply uniform bioinformatic pipelines and directly compare cohorts. On the analysis side, more sophisticated statistical techniques should be employed to adjust for confounders (e.g., multivariate models, batch effect corrections, and the inclusion of random effects for household or cohort). As an example, one might include diet score,^59^ BMI, HIV status, and antibiotic history as covariates in any analysis linking microbiome features to TB—something not uniformly done in older studies. Embracing multiomics also presents a standardization challenge: different omics data have different noise characteristics, and it is crucial to use robust methods for data integration to avoid spurious correlations. Harmonizing how studies report their results (perhaps adopting the STORMS reporting guidelines proposed for microbiome studies in disease^37^ will facilitate concise and complete reporting in microbiome studies).

### Causal inference and mechanistic studies

Most human TB–microbiome studies are observational, identifying associations but not causation. Stronger study designs are needed to determine whether microbiome changes influence susceptibility to TB or are a consequence of the disease. Longitudinal studies, such as tracking latent TB cohorts, could reveal whether the baseline microbiota can predict progression. Interventional models—antibiotic-treated, germ-free, or fecal transplant in mice—show that microbiota depletion worsens TB outcomes.^55^ Already, mice treated with antibiotics to deplete the gut microbiota show worse TB outcomes,^55^ supporting a causal protective role. “Humanized” mice colonized with TB patient vs. healthy donor microbiota could test transmissible susceptibility. In humans, probiotic or dietary trials could assess impacts on immunity and outcomes, where faster sputum clearance or reduced inflammation would imply causality. Mendelian randomization,^57^ offers another tool, using genetic proxies for microbiome traits. Mechanistic work should dissect pathways, e.g., how butyrate-producing bacteria enhance macrophage Mtb killing. Multiomics can identify candidate metabolites for in vitro testing with immune cells. This shift from correlation to experimental validation will provide the rationale for microbiome-targeted therapies.

### Expanding scope: mycobiome, virome and beyond 16S

Future TB microbiome research should broaden its scope beyond bacteria. The gut mycobiome (fungal community) and virome (viruses, including bacteriophages) are parts of the mucosal ecosystem that have been relatively neglected. Initial studies suggest that the mycobiome is perturbed in TB; for example, in a comparative cross-sectional study, people with TB were found to have a perturbed gut mycobiome marked by significantly reduced fungal diversity and increased abundance of *Candida* and *Saccharomyces* species compared to healthy controls.^5^^,^^60^ Fungal products (such as β-glucans) can influence immune responses, so changes in fungi might also matter for TB immunity. Likewise, phages that infect gut bacteria could impact which bacteria thrive under TB treatment. Going forward, integrated analyses of bacteria, fungi, and viruses (possible with metagenomic sequencing) will provide a more complete picture of the microbiome changes in TB.

### Special populations (pediatric TB, extrapulmonary TB, asymptomatic TB)

Most studies have focused on adult pulmonary TB. Pediatric TB is an important context: the gut microbiome of children is still developing, highly sensitive to diet, and potentially more vulnerable to long-lasting disruption from antibiotics. Malnutrition and TB often coexist in children; it would be valuable to study how nutritional rehabilitation and microbiome restoration interplay in pediatric TB outcomes.^8^ Similarly, extrapulmonary TB (such as TB meningitis or intestinal TB) might have unique microbiome considerations. For example, recent studies characterizing the site-of-disease microbiome in tuberculous lymphadenitis and pericarditis have shown that these niches are not microbially homogeneous but rather display distinct microbial “lymphotypes” or pericardial profiles associated with HIV status, disease severity, and enrichment of pathways such as short-chain fatty acid metabolism.^61^^,^^62^

Intestinal TB could also be directly influenced by the gut microbiota composition, given its gastrointestinal localization; indeed, one study suggested that gut microbiome profiles could help distinguish intestinal TB from other inflammatory bowel conditions.^63^ Going forward, including these special cases will broaden our understanding and perhaps reveal stronger microbiome signals—for instance, the gut microbiome might be even more relevant in intestinal TB pathogenesis.

### Therapeutic development and clinical trials

To move towards clinical translation, interventional trials are needed to test microbiome-based strategies. This is a clear future direction: small Phase II trials could investigate whether adding a certain probiotic to standard TB therapy improves patient weight gain or reduces gut inflammation (measured by fecal calprotectin, for instance). If successful, larger trials could measure hard outcomes such as sputum conversion rates or the incidence of hepatotoxicity. There is also interest in whether post-TB lung disease (the chronic lung impairment many TB survivors have) can be ameliorated by microbiome modulation—perhaps an anti-inflammatory microbiome could reduce fibrosis or chronic obstructive changes. Trials to address this issue would need long-term follow-up. Another futuristic idea is microbiome-derived pharmaceuticals: identifying a metabolite from a commensal that has anti-TB properties or immunomodulatory effects and developing it as a drug. For example, if indolepropionic acid (a microbial metabolite) proves to increase host TB defenses, one could test giving it as a supplement.^43^

These strategies align with the growing global focus on host-directed therapies for TB,^64^ and the microbiome makes a strong candidate for inclusion in this pipeline, given its ability to modulate immunity, inflammation, and drug metabolism. Such translational leaps will require a pipeline from discovery to preclinical testing to clinical trials.

### Collaboration and interdisciplinary research

The complexity of TB–microbiome interactions means that future progress will rely on collaboration across disciplines—microbiologists, TB clinicians, immunologists, bioinformaticians, and even social scientists (for diet/lifestyle data) need to work together. Large cohort studies such as Stool4TB^60^^,^^65^ incorporate microbiome analyses alongside detailed clinical phenotyping; similar integrative projects should be encouraged. International collaborations could enable studying how the microbiome–TB link might differ in high-TB burden settings (with more environmental mycobacterial exposure, for instance) vs. low-burden settings. There is also a need for capacity building in low-income countries to perform microbiome research, as these are the regions where TB is most prevalent and local factors will influence the microbiome. Future studies should be designed with community engagement, particularly when diet or nutritional interventions are considered, to ensure feasibility and cultural acceptability.

### Knowledge gaps

**Figure 5.**
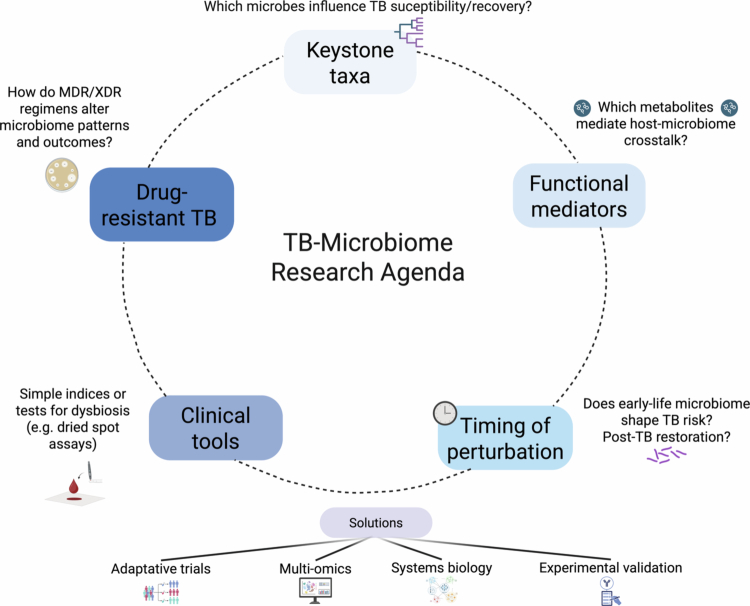
Knowledge gaps and research priorities in TB–microbiome research. Unanswered questions include: (1) identifying keystone microbial species or consortia that influence TB susceptibility or recovery; (2) defining microbial metabolites that mediate host–microbiome crosstalk, such as short-chain fatty acids or tryptophan catabolites; (3) clarifying the timing and persistence of microbiome perturbations, from early-life influences on TB risk to restoration after treatment; (4) developing practical microbiome-based tools for clinical use, such as simplified metabolite indices or dried blood spot assays; and (5) characterizing how multidrug-resistant TB regimens reshape microbial communities and impact outcomes. Addressing these gaps will require adaptive clinical trials, systems biology and multiomics integration, and experimental validation to move from observational associations to causal and translational insights.

Some of the key questions going forward are summarized in Figure 5. Addressing these factors will likely require novel study designs, such as adaptive trials that can test multiple interventions, or systems biology models that integrate multiomics data to predict outcomes, which can then be experimentally validated.

In summary, the future of TB–microbiome research is bright but challenging. By tackling confounders, standardizing methodologies, and focusing on mechanistic and translational studies, the field can progress from observational association to evidence-based applications. As one Lancet Respiratory Medicine review aptly stated, the goal is to reach a stage where microbiome insights can be “defining the clinical research agenda” for TB and be harnessed for patient benefit.^15^ This goal will require patience and rigor, but the potential rewards—improved TB diagnostics, adjunct therapies to shorten treatment or reduce suffering, and perhaps even preventative strategies leveraging the microbiome—are well worth the concerted effort.

## Conclusion

A decade of research has firmly established that TB does not occur in isolation but within the complex ecosystem of the human microbiome. While disruptions to the gut and lung microbiota in TB are increasingly documented, interpreting these changes demands rigorous attention to confounders, methodological standardization, and a shift from descriptive to functional analyses. This review highlights the need to move beyond compositional surveys and adopt multiomics strategies to identify mechanistic links between microbiome alterations and TB pathogenesis or treatment outcomes.

The translational potential of microbiome science in TB is now tangible: microbiome-derived biomarkers could improve diagnosis or risk stratification; monitoring microbiome recovery may predict treatment success or relapse; and adjunctive therapies—such as nutritional or probiotic interventions—will require methodologically harmonized, causally informative, and context-specific research across diverse TB-endemic settings.

The field stands at a turning point: from observational association to mechanistic understanding and clinical innovation. To accelerate progress, we must foster interdisciplinary collaboration, build capacity in high-burden regions, and establish international standards for TB–microbiome research. With rigorous science and coordinated effort, integrating microbiome insights into TB care could transform both our biological understanding and the clinical management of this global disease. Ultimately, recognizing the host as an ecosystem—not just a battleground between pathogen and immunity—opens new frontiers in precision TB medicine.

## Data Availability

No primary data were generated. The review is based on analysis and interpretation of previously published data, as referenced throughout the manuscript.
